# Artificial Weathering Resistance Test Methods for Building Performance Assessment of Profiles Made of Natural Fibre-Reinforced Polymer Composites

**DOI:** 10.3390/ma15010296

**Published:** 2021-12-31

**Authors:** Ewa Sudoł, Ewelina Kozikowska, Ewa Szewczak

**Affiliations:** 1 Construction Materials Engineering Department, Instytut Techniki Budowlanej, 00-611 Warsaw, Poland; e.kozikowska@itb.pl; 2 Group of Testing Laboratories, Instytut Techniki Budowlanej, 00-611 Warsaw, Poland; e.szewczak@itb.pl

**Keywords:** artificial weathering testing in civil engineering, construction profiles, natural fibre-reinforced polymer composites, building performance assessment, microstructure analysis, mechanical properties

## Abstract

A growing popularity of profiles made of natural fibre-reinforced polymer composites in civil engineering encourages determining test methods relevant for building performance assessment. Weathering resistance is among the key aspects that condition the durability of building structures. The paper includes a comparative analysis of two artificial weathering resistance test methods. Polyvinyl chloride and wood flour composite profiles were tested. They were subjected to UV and spraying (X-exposure) and UV, spraying and wetting by condensation (F-exposure), both at different exposure times. The influence of the applied weathering procedures on the composite’s microstructure and its mechanical characteristics were analysed. No changes in the microstructure of brittle fractures were observed. However, surface morphology changes were revealed, noticeably greater following X-exposure than F-exposure. F-exposure exerted significant influence on the mechanical properties of brushed profile, including, but not limited to, flexural modulus. Whereas X-exposure exerted more influence on the mechanical properties of non-brushed profile.

## 1. Introduction

Natural fibre-reinforced polymer composites (NFPC) have been used in many industry branches for a number of years. Nowadays, it is hard to imagine the medical, automotive, aerospace and shipyard sectors and civil engineering without them [1,2,3,4]. NFPC, as well as carbon nanotubes-reinforced polymer composites rapidly growing [5].

**Figure 1:**
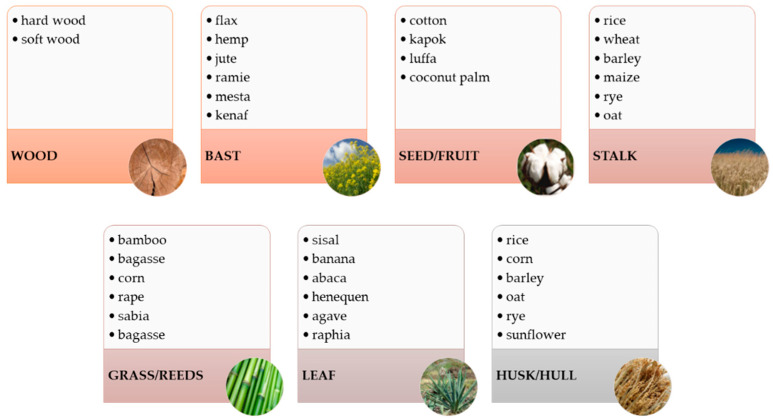
Types of plant fibres used in NFPC.

Natural fibres nearly completely replaced synthetic fibres in polymer composites [6]. Nowadays, NFPCs include mainly lignocellulosic fibres obtained from different tree, grass and crop species. Their biodegradability, source renewability, low density at high strength and elasticity, and low cost and neutrality for humans and tools have been appreciated [7]. The fibres are obtained from hard and soft tissues (Figure 1)—wood, stalks, seeds, leaves, fruit, phloem, husks and shells being waste from agricultural production [2,7].

The most popular NFPC matrices include polypropylene (PP), high-density polyethylene (HDPE), polyvinyl chloride (PVC), and sometimes polystyrene (PS) [7,8,9]. Matrix selection depends on the composite’s intended use [4]. Some matrices are made of biodegradable polymers, e.g., polyglycolic acid (PGA) and polyhydroxyalkanoates (PHA) [9].

**Figure 2:**
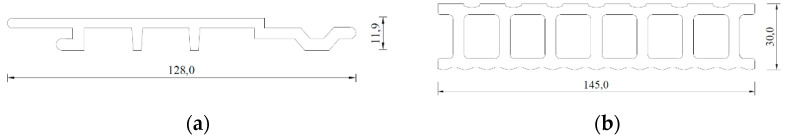
Sample NFPC building profiles for (**a**) facades, (**b**) floors. The dimensions are given in mm.

**Figure 3:**
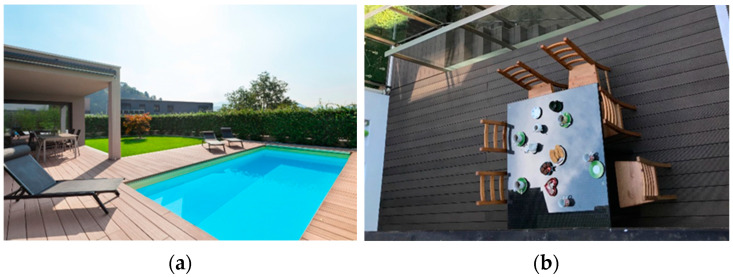
Sample application of NFPC profiles in civil engineering for outdoor floors: (**a**) at a swimming pool, (**b**) on the terrace.

Civil engineering applications are dominated by NFPC with PVC or HDPE matrix [4,6]. They are used in solid (Figure 2a) or cellular (Figure 2b) profiles intended for outdoor floors—on terraces and swimming pools (Figure 3) and ventilated façade cladding [10,11,12]. NFPC profiles are also employed in platforms, passages, landscape architecture and wet rooms [3,4,5,6].

The fitness of NFPC profiles for civil engineering applications, similarly to other construction materials, should be assessed according to the sustainable development concept, based on the usability criterion, by determining a collection of key features for the particular application [13]. The assessment is carried out from the angle of the product’s influence on a building structure’s fulfilling the seven essential requirements [14], according to the regulation of the European Parliament and the Council (EU) No. 305/2011 (CPR) [15]. The seventh essential requirement concerning the Sustainable use of natural resources states that building structures need to be designed and made so that natural resources are used sustainably and ensure the durability of building structures. Fulfilling the building structure’s durability criterion depends on the construction products’ resistance to operating factors, including the environmental ones [16,17]. The aspect of resistance to environmental conditions has special significance for outdoor products, such as terrace floors and façade cladding, which are directly exposed to long-term sun radiation, water impact, temperature changes and microbiological factors [12,18]. So far the weathering resistance test methods for building performance assessment of profiles made of natural fibre-reinforced polymer composites have not been standardized.

Ensuring efficient interaction between lignocellulosic fibres and the polymer matrix is among the key challenges for natural fibre-reinforced polymer composites to achieve proper resistance to environmental factors [4,9]. The hydrophilic nature of the fibres makes them swell in an aqueous environment, which results in cracks formed in the hydrophobic polymer matrix [18]. As a result, the interaction between lignocellulosic fibres and the polymer deteriorates. Insufficient adhesion at the phase border leads to decreased mechanical parameters [9,19]. Hence, the fibres’ surface is modified to improve the interphase interaction by increasing the fibres’ wettability and reducing water absorption [4,9]. The most popular chemical methods involve employing substances whose particles react with cellulose hydroxy groups and introduce new groups linked with the polymer matrix’s functional groups [20]. Similar treatments are used in the case of carbon nanotube reinforcement [21]. The number, shape, size and distribution of the fibres also affects the NFPC characteristics [4,7,8]. Proper scattering of fibres in the matrix promotes interphase adhesion by reducing voids and ensuring the fibres’ surrounding by the matrix [2,4,21].

NFPC products are susceptible to sunlight [18,22]. Their exposure to UV light was discovered to contribute to a more significant decrease in the mechanical properties than exposure to microorganisms and high temperatures [23,24,25,26,27,28]. Tests on NFPC products’ resistance to sunlight are typically carried out with accelerated methods, using laboratory light sources [27,28,29,30,31,32,33,34,35,36]. The application of accelerated methods involving product exposure to the relatively short but intensive impact of a factor or a set of service factors is standard for construction fitness assessment procedures. The impacts are selected according to the product’s material characteristics, including the product’s application scope, to simulate best the processes that occur during the product’s use in real conditions [17]. As shown by previous studies, light and water impact cycles are the most burdensome exposure sequence in NFPC’s accelerated weathering [32,33,34,35,36,37,38]. NFPC degradation progresses then much faster and more intensively than in the case of exposure to sunlight only [30]. In the wetting phase, the polymer matrix particles damaged as a result of UV impact are washed out, and successive ones are exposed [22], but hydrophilic lignocellulosic fibres swell too, which leads to reduced interphase adhesion, as was mentioned before [18,19]. As was already determined [34], the light exposure and spraying cycles cause much more intensive destruction of profiles whose surfaces were mechanically treated before than surfaces non-treated after extrusion. Composites with a higher share of lignocellulosic fibres on the surface, exposed during planing, show a higher drop in the flexural modulus [31,34]. NFPC was discovered to degrade faster than the polymer used as a matrix. At the initial weathering exposure stage, a pure polymer may be subjected to further cross-linking, while this property is physically limited in a composite by the filler [35]. The exposure time matters as well. The longer it is, the greater degradation occurs [34,35,36]. Light exposure reduces the mechanical properties and changes the NFPC products’ colour [30,37].

Analysing previous studies on artificial weathering resistance of NFPC products addressed for civil engineering, it can be observed that different light sources are used in the exposure procedures, with diversified exposure sequence, including the dry and wet phase length and wetting method [34,35,36,37,39,40]. This paper contains a comparative analysis of the two most common methods used for construction products to determine the most relevant building performance assessment for natural fibre-reinforced polymer composite profiles. So far the comparative analysis of artificial weathering resistance test methods has not been performed. Tests were carried out for PVC and wood flour composite profiles as one of the most popular in civil engineering [10,11,26]. The influence was analysed of the applied exposure procedure on the changes in the composite microstructure and mechanical properties. A comparative exposure was performed, including the following:

- exposure to light emitted by xenon lamps (X-exposure) combined with alternate short spraying, with diversified exposure time,
- wetting through long-term condensation and then exposure to light emitted by fluorescent lamps (F-exposure), with diversified exposure time.

The influence was evaluated of the performed exposures on the usable surface’s morphology and microstructure of brittle fractures, flexural strength, flexural modulus and impact strength.

## 2. Materials and Methods

### 2.1. Profiles

Commercial cellular profiles intended for outdoor floors were used for the tests. The profiles were made of PVC matrix composite with fine lignocellulosic fibre filler (wood flour) and plastifiers and modifiers as additives. The filler was recycled wood industry waste. The composite’s formula is the manufacturer’s trade secret and has not been revealed. The profiles were extruded in a plastic processing facility.

**Figure 4:**
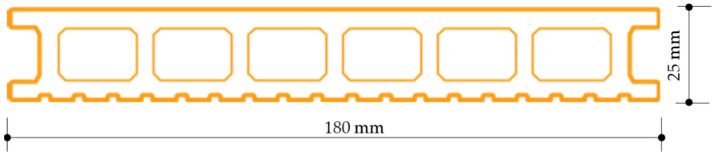
Shape of the profiles used in the tests.

The profiles were 180 mm wide, 25 mm high, the front walls were 5 mm thick, and the chambers were 22 mm wide. The profiles had two usable surfaces: one grooved and one plane (Figure 4). As a standard, grooved and plane surfaces of construction profiles are mechanically treated (brushing) to provide a wood-like texture effect. Profiles with a standard usable brushed surface (SZ) and profiles with a non-brushed usable surface (NSZ)—for comparison—were used in the study.

### 2.2. Weathering Exposure

The first weathering procedure (F-exposure) was carried out in UV Test apparatus (Atlas, Linsengericht, Germany) featured with 1A type (UVA-340) fluorescent lamps according to EN 16474-3 [41], emitting light in the wavelength range of 300 to 400 nm, with the maximum emission at 343 nm (Table 1). The exposure procedure complied with EN 927-6 [42]. The samples were exposed to cycles composed of a long condensation phase, followed by exposure to UV lamps, with the radiation intensity of 0.89 W/m^2^ measured at 340 nm wavelength, with alternated wetting cycles (water spraying) (Table 2).

**Figure 5:**
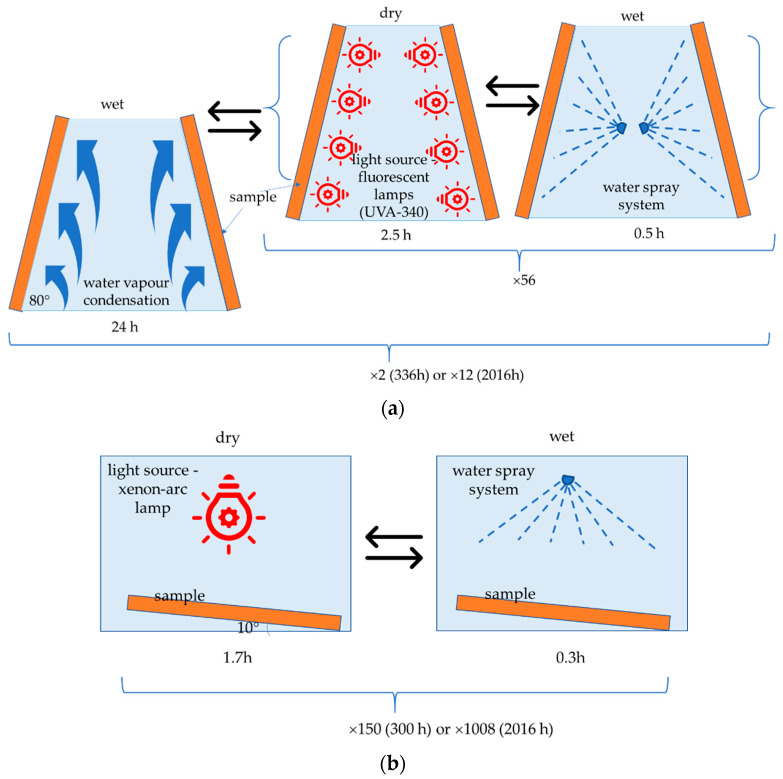
Weathering exposure procedure flowchart: (**a**) F-exposure, (**b**) X-exposure.

Samples cut out from flat usable surfaces of brushed (SZ), and non-brushed (NSZ) profiles were exposed. The samples were 300 mm long, and the profiles’ full width (180 mm) was maintained. During the exposure, the samples were arranged at ca. 80° angle, allowing free draining of water (Figure 5a). The exposure lasted 336 h—SZ-F-336 and NSZ-F-336 series, and 2016 h—SZ-F-2016 and NSZ-F-2016 series (Table 2).

The other weathering procedure (X-exposure) was performed in SunTest apparatus (Atlas, Linsengericht, Germany) featuring a xenon-arc lamp with a quartz shell, according to EN 1647-2 [43], which emits light from less than 270 nm in the ultraviolet range through visible spectrum up to IR, whereby a daylight filter was used, eliminating shortwave UV radiation (Table 3). Exposure was carried out according to EN ISO 4892-2 method A [44]. The cycles included exposure to light with the radiation intensity of 60 W/m^2^, measured in the band wavelength of 300–400 nm, combined with exposure to high temperature, and followed by water spraying (Table 2).

**Table 1:** Relative spectral intensity of radiation for UVA 340 fluorescent lamps [39] used in the UV test apparatus.

Spectral Pass Band	Minimum	CIE No. 85:1989, Table 4	Maximum
(λ = Wavelength in nm)	%	%	%
λ < 290	-	-	0.1
290 ≤ λ ≤ 320	5.9	5.4	9.3
320 < λ ≤ 360	60.9	38.2	65.5
360 < λ ≤ 400	26.5	56.4	32.8

**Table 2:** Weathering exposure.

Test Series Designation	Total Exposure Time	Exposure Method/Light Source	Number of Cycles	Exposure during the Cycle
SZ-F-336 NSZ-F-336	336 h	**F-exposure** according to EN 927-6/ UVA340 fluorescent lamps	2	24 h of wetting through condensation at T45 ± 3 °C 168 h of alternate light exposure and water spraying cycles, in the following sequence: - 2.5 h of exposure to UVA−340 lamps, radiation intensity: 0.89 W/m^2^ (340 nm), BST 60 ± 3 °C, - 0.5 h water spraying without UV exposure, spraying intensity 6−7 L/min.
SZ-F-2016 NSZ-F-2016	2016 h		12	
SZ-X-300 NSZ-X-300	300 h	**X-exposure** according to EN ISO 4892-2/ xenon-arc lamp with daylight filter	150	1.7 h of irradiation with lamps, radiation intensity: 60 ± 2 W/m^2^ (300–400 nm), BST 65 ± 3 °C, CHT 38 ± 3 °C, RH 50 ± 10%, 0.3 h of water spraying without UV exposure
SZ-X-2016 NSZ-X-2016	2016 h		1008	

**Table 3:** Relative spectral intensity of radiation for a xenon-arc lamp with daylight filter [43] used in SunTest apparatus.

Spectral Pass Band	Minimum	CIE No. 85:1989, Table 4	Maximum
(λ = Wavelength in nm)	%	%	%
λ < 290	-	-	0.15
290 ≤ λ ≤ 320	2.6	5.4	7.9
320 < λ ≤ 360	28.2	38.2	39.8
360 < λ ≤ 400	54.2	56.4	67.5

**Table 4:** Relative differences between the values of the mechanical properties of SZ and NSZ samples, calculated according to Formula (6), %. Statistically insignificant differences are highlighted in grey.

*σ_f_*—Flexural Strength	*E_f_*—Modulus of Elasticity	*a_cU_*—Charpy Impact Strength
10.2	0.1	9.6

A brushed (SZ) and non-brushed (NSZ) flat usable surfaces were exposed. The samples’ length ranged from 100 to 300 mm, and their width and thickness corresponded to the profile’s dimensions. During the exposure, the samples were arranged horizontally, maintaining a ca. 10° slope to allow free draining of water (Figure 5b). The exposure lasted 300 h for the SZ-X-300 and NSZ-X-300 series and 2016 h for the SZ-X-2016 and NSZ-X-2016 series (Table 2).

Deionised water with pH 5.0 ± 7.5 and electric conductivity under 2 μS/cm measured at 25 °C were used for wetting in both weathering procedures. 

### 2.3. SEM Analysis

The microstructure of composite profiles was examined with Sigma 500 VP cold-field emission scanning electron microscope (Carl Zeiss Microscopy GmbH, Köln, Germany), which allows reaching a high resolution at a low accelerating voltage. The tests were carried out at the accelerating voltage of 10 KeV inductive electron beam, using an SE detector on samples coated (sprayed) with a gold film.

At the first stage, the microstructure of brittle fractures obtained at 23 °C was observed. The observations covered samples cut out from brushed (SZ) and non-brushed (NSZ) profiles in their original condition and following X-exposure lasting 2016 h (SZ-X-2016 and NSZ-X-2016 series). The procedure was selected because it is expected to cause the most significant changes in the NFPC structure [45]. Observations were carried out at 500× and 20,000× magnification. At the second stage, the observations covered the usable surface microstructures in NSZ profiles in their original condition, following F-exposure (NSZ-F-336 and NSZ-F-2016 series) and X-exposure (NSZ-X-300 and NSZ-X-2016 series), at 500× magnification. The observations were not carried out for brushed profiles because of the high roughness of the usable surface, which made SEM examinations impossible.

### 2.4. Testing Mechanical Properties

Mechanical properties were tested on samples obtained from brushed (SZ) and non-brushed (NSZ) profiles in their original condition and following a short- and long-term F-exposure and X-exposure (Table 2). The flexural strength, flexural modulus and impact strength were tested.

**Figure 6:**
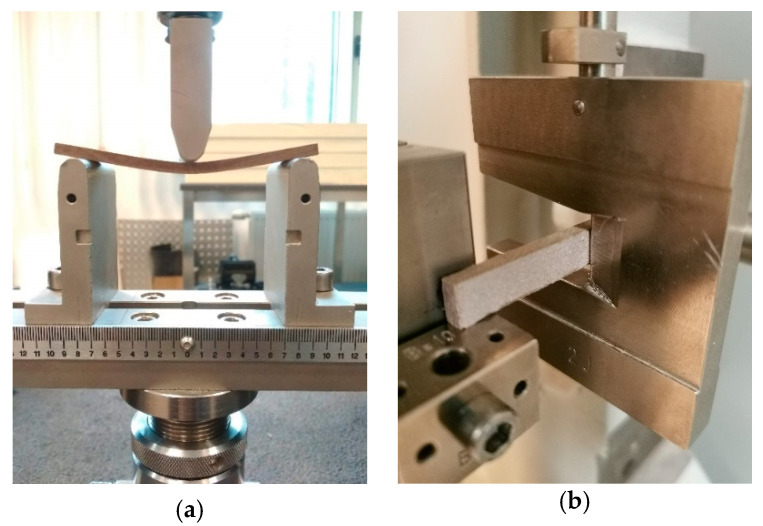
Testing mechanical properties: (**a**) flexural strength, (**b**) impact strength.

The flexural modulus was also tested according to EN ISO 178 [46], using a class 1 strength testing machine (Instron, Darmstadt, Germany). Three-point bending was performed according to EN ISO 178 [46], using samples sized 15 × 100 × 5 mm, cut out from the central part of the profile’s front wall, parallel to vertical ribs (Figure 4). Supports with a 5 mm radius were used, spaced every 80 mm, corresponding to 16-times sample’s thickness and a 5 mm radius pressing element placed in the middle of the span. The samples were freely supported (Figure 6a). The load was applied to the front surface at a constant rate of 5 mm/min. until destruction. Flexural strength *σ_f_* was calculated according to (1) and expressed in N/mm^2^. Twelve samples were tested in each series, giving a total of one hundred and twenty samples tested in the study.

(1) $$\sigma_{f}=\frac{3FL}{2bh^{2}}$$

where: *F*—maximum force, in N; *L*—support spacing, in mm; *b*—sample’s width, in mm; *h*—sample’s thickness, in mm.

The flexural modulus was also tested according to EN ISO 178 [46], using a class 1 strength testing machine (Instron, Darmstadt, Germany), in conditions identical to flexural strength tests. A load-deflection curve was recorded during bending in a linearly elastic range, including the force and deflection values corresponding to strain ε*_f_*_1_ = 0.0005 and ε*_f_*_2_ = 0.0025. The *f*_1_ and *f*_2_ deflection values were calculated according to Formula (2).

(2) $$f_{1}=\frac{\varepsilon_{f1}L^{2}}{6h};\;f_{2}=\frac{\varepsilon_{f2}L^{2}}{6h}$$

where: *L*—spacing of supports, in mm; *h*—sample’s thickness, in mm.

The force values recorded when ε*_f_*_1_ and ε*_f_*_1_ strain occurred were used for determining the values of σ*_f_*_1_ and σ*_f_*_2_ normal stress. The *E_f_* modulus was calculated according to (3) and expressed in N/mm^2^. Twelve samples were tested in each series, giving a total of one hundred and twenty samples tested in the study.

(3) $$E_{f}=\frac{\sigma_{f2}-\sigma_{f1}}{\varepsilon_{f2}-\varepsilon_{f1}}$$

where: σ*_f_*_1_, σ*_f_*_2_—maximum normal stress corresponding to *f*_1_ and *f*_2_ stress determined according to (2).

The impact test was carried out with Charpy impact pendulum (ZwickRoell, Ulm, Germany) according to EN ISO 179-1 [47]. The samples used in the test had no notch, were sized 10 × 80 × 5 mm, cut out from the central part of the profile’s front wall, parallel to the vertical ribs. The sample was freely resting on supports spaced at 62 mm and then hit with a 2J impact pendulum (Figure 6b). The load was exerted on the front surface. Charpy impact strength *a_cU_* was calculated according to (4) and expressed in kJ/m^2^. Eight samples were tested in each series, giving a total of eighty samples tested in the study.

(4) $$a_{cU}=\frac{E_{c}}{h\cdot b}\cdot 10^{3}$$

where: *E_c_*—energy absorbed by breaking the test specimen, in J; *h*—sample’s thickness in mm; *b*—sample’s width, in mm.

### 2.5. Analysis of the Statistical Difference in the Mechanical Properties Test Results

The changes in the tested materials’ mechanical properties were analysed based on the characteristics’ differences after F-exposure and X-exposures. Since in most cases, the differences between the results before and after the exposure were relatively low compared to the results’ variability in the groups, the statistical significance of the differences was analysed with a one-way analysis of variance (ANOVA F-test).

The difference in the given mechanical property before and after weathering (Δ*Y*) was calculated with the following equation:

(5) $$\Delta Y=100\%\cdot \frac{Y\left(T_{j}\right)-Y\left(T_{i}\right)}{Y\left(T_{i}\right)}$$

where: *T_i_*, *T_j_*—ageing times used; *Y*(*T_i_*)—mean value of the given mechanical property after weathering for *T_i_*, *Y*(*T_j_*)—mean value of the given mechanical property after weathering for *T_j_*.

Taking into account that two sample series—obtained from brushed (SZ) and non-brushed (NSZ) usable surface of the profiles—were subjected to mechanical properties tests before and after weathering exposure, an analysis of the exposure influence on the properties of interest was preceded by an assessment of the differences between the properties of SZ and NSZ samples in their original condition. The following formula was used for calculating the relative difference:

(6) $$\Delta Y=100\%\cdot \frac{Y_{SZ}-Y_{NSZ}}{Y_{SZ}}$$

where: ΔY—difference between the mechanical properties of material *Y* with brushed *SZ* (*Y_SZ_*) and non-brushed NSZ (*Y_NSZ_*) surface.

The statistical significance of the differences was analysed with ANOVA F-test. The results are summarised in Table 4. No surface treatment influence was observed only for the modulus of elasticity. For flexural strength, the relative difference between the values obtained for SZ and NSZ samples amounted to 10.2%, while for the Charpy impact strength it was 9.6%. Both characteristics were higher for SZ than for NSZ samples. The exposure impact on all analysed mechanical properties was assessed separately for each surface type because of the statistically significant difference in the flexural strength and Charpy impact strength tests for SZ and NSZ surface samples.

## 3. Results and Discussion

### 3.1. Microstructure Analysis

**Figure 7:**
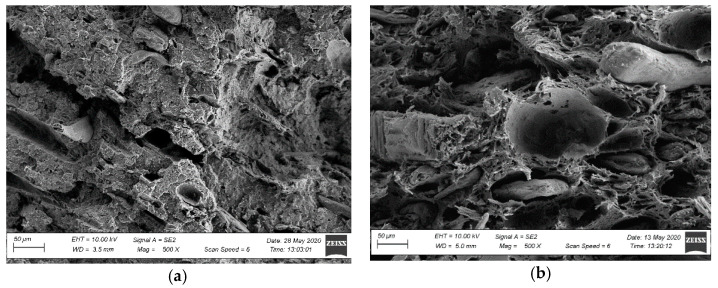
Microstructure of the profile’s fracture surface in the original condition: (**a**) SZ profile, magnification: 500×, (**b**) NSZ profile, magnification: 500×.

**Figure 8:**
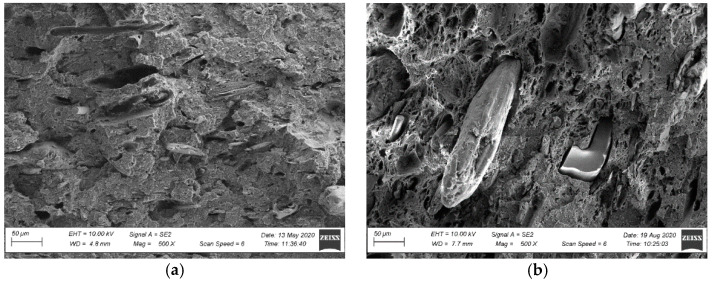
Microstructure of the profile’s fracture surface after weathering: (**a**) SZ-X-2016 series profile, magnification: 500×, (**b**) NSZ-X-2016 series, magnification: 500×.

**Figure 9:**
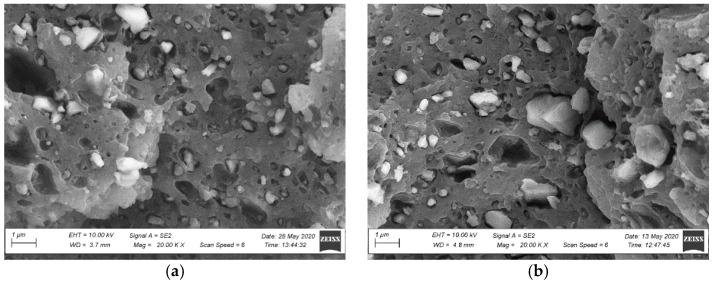
Microstructure of the profile’s fracture surface in the original condition: (**a**) SZ profile, magnification: 20,000×, (**b**) NSZ profile, magnification: 20,000×.

**Figure 10:**
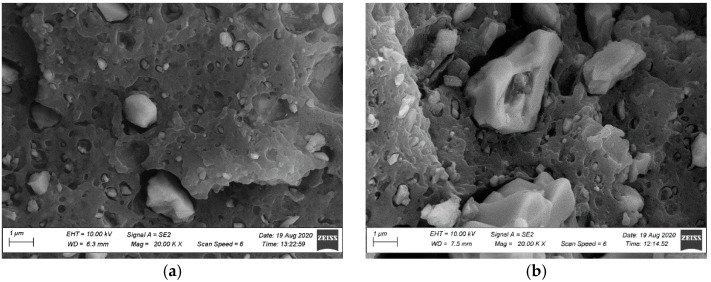
Microstructure of the profile’s fracture surface after weathering: (**a**) SZ-X-2016 series profile, magnification: 20,000×, (**b**) NSZ-X-2016 series profile, magnification: 20,000×.

The observations of the brittle fracture microstructures helped evaluate only the dispersion rate of a filler in a polymer matrix. The composite’s observed structure can be considered inhomogeneous [47,48]. Numerous wood flour clusters were discovered, forming combinations of fibres and plates with diameters ranging from 50 µm to 100 µm (Figure 7 and Figure 8). Because a fracture in a composite occurs typically in the sample’s most weakened areas, material defects in the form of pores and voids became visible at the fracture, being a testimony to the plates and wood fibres being pulled out from the polymer matrix [48] (Figure 7 and Figure 8). Further analysis of the brittle fractures’ microstructure revealed the presence of other fillers’ clusters, most likely being mineral fillers (talc or chalk) and relatively regular shape and size not exceeding 1 µm. They were generally well dispersed in the polymer matrix (Figure 9 and Figure 10), but some cluster sizes from 5 µm to 10 µm (Figure 10b) were also discovered. The performed SEM analysis of brittle fractures did not reveal microstructural differences in the material in its original condition compared to the material after X-exposure for 2016 h (SZ-X-2016 and NSZ-X-2016 series). The data collected in the brittle fracture analysis, revealing the microstructure at the material cross-section, can suggest that the material’s inner structure did not change under the influence of the applied weathering procedure.

**Figure 11:**
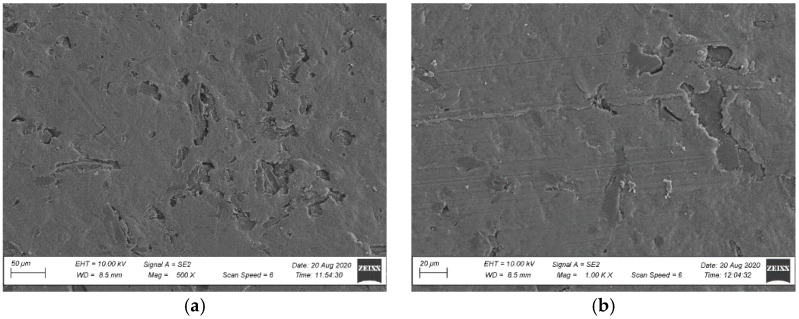
Microstructure of NSZ profile’s surface in the original condition (**a**) magnification: 500×, (**b**) magnification: 1000×.

**Figure 12:**
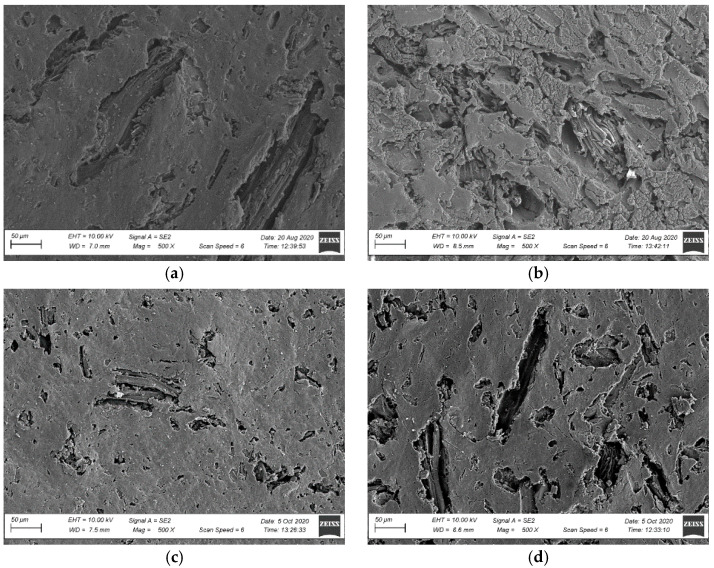
Surface microstructure after weathering, magnification: 500× (**a**) NSZ-X-300 series, (**b**) NSZ-X-2016 series, (**c**) NSZ-F-336 series, (**d**) NSZ-F-2016 series.

An SEM surface analysis was carried out, taking into account the fractures’ surface microstructure analyses and bearing in mind that the profiles’ usable surface was directly exposed. The tests covered only the non-brushed profiles because of brushed profiles’ high surface roughness, which prevented their observations. An analysis of NSZ samples’ surface microstructure in the original condition revealed a uniform coating of the fibres with polymer (Figure 11). The surface was relatively smooth and uniform, characteristic of extruded NFPC profiles [48,49]. No exposed wood fibres were observed. Following the profiles’ X-exposure, significant changes in the surface morphology were observed already after 300 h. The microscopic image revealed melting of the polymer’s outermost layer, exposing the surfaces of fillers not wetted with the polymer, taking the form of large plates and wood fibre clusters (Figure 12a). Extending the exposure time to 2016 h significantly aggravated the top layer’s degradation. Highly non-homogenous surface topography with molten areas was observed [50]. The revealed microstructure contained agglomerated wood fibres (Figure 12b).

F-exposure also contributed to the changes in the surface morphology. The microstructure changes were reported after 336 h of exposure (NSZ-F-336 series), and minor molten areas in the polymer’s outermost layer became visible, exposing the filler’s surface (Figure 12c). Still, the changes are noticeably more minor than those reported for samples after X-exposure for 300 h (Figure 12a). The F-exposure time extension to 2016 h aggravated the profile’s outermost layer, making the filler much more visible (Figure 12d). It should be emphasised that the degradation rate of NSZ-F-2016 series samples was significantly lower than the degradation rate of samples after X-exposure for the same exposure duration (NSZ-X-2016 series).

Summing up the results of microstructural tests, it can be concluded that the applied weathering procedures performed with laboratory light sources did not affect the composite’s internal structure. No differences that could be considered microstructure changes were observed in the brittle fracture analysis [48,49]. Under UV-irradiation influence the surface layers between PVC matrix and wood fibres became more brittle. Due to these factors create additional stresses at the interface of the components, causing development of the cracks on the weathered surface of the samples [49]. Exposing the profile to light emitted by fluorescent lamps (F-exposure) and xenon lamps (X-exposure) caused significant surface degradation. The surface morphology analysis revealed molten areas in the polymer matrix’s outermost layer, exposing the surface of lignocellulosic fibres. Extended exposure aggravated the degradation of the profiles’ usable layer, which corresponds to the literature data [45,50]. Weathering impacts exerted with a xenon lamp affected the surface properties more significantly than F-exposure for the same exposure time. The above can be explained by the differences between relative spectral intensity of radiation for UVA 340 fluorescent lamps (F-exposure) and relative spectral intensity of radiation for a xenon-arc lamp (X-exposure). It is supposed that wavelengths rays between 360 nm and 400 nm is the most important factor causing photodegradation to some organic substances such as PVC. F-exposure contains about 26% wavelengths rays between 360 nm and 400 nm (Table 1) while X-exposure contains about 54% (Table 3). Surface damage after X-exposure was more intensive and vast than after F-exposure.

### 3.2. Mechanical Properties

**Figure 13:**
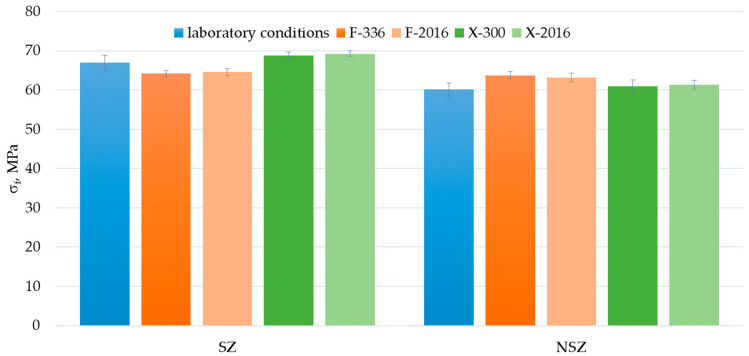
Flexural strength test results of brushed (SZ) and non-brushed (NSZ) samples in basic state (laboratory conditions), after F-exposure for 336 h (F-336) and 2016 h (F-2016) and after X-exposure for 300 h (X-300) and 2016 h (X-2016). The error bands represent the standard deviation (series size n = 12).

An analysis of the results suggests that the analysed material’s flexural strength in the original condition was 60 ÷ 67 MPa; 63 ÷ 64 MPa after F-exposure, and 61 ÷ 64 MPa after X-exposure (Figure 13). These values are similar to those obtained for construction profiles made of wood fibre-reinforced composites with PE matrix and PVC matrix with rice husk fibre, for which the original condition values amounted to 71 MPa and 67 MPa, respectively [51]. They exceed the test results on composites with recycled high-density polyethylene matrix and rice husk fibre filler, which reached the flexural strength of 25 MPa for the filler content of 50% and 38 MPa for the filler content of 80% [28]. Still, they are lower than the results for composites with polymer matrix reinforced with sycamore, sisal or bamboo fibres, whose flexural strength ranged from 100 MPa to 134 MPa [52].

**Figure 14:**
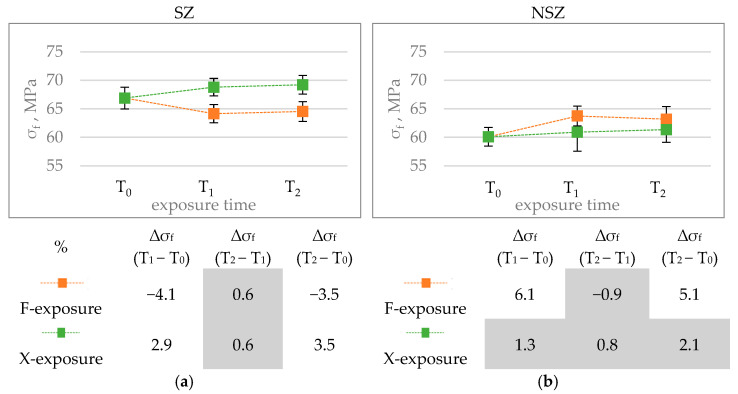
Diagrams showing the differences in the sample’s flexural strength σ_f_, MPa, after F-exposure and X-exposure for time T_1__and_ T_2_: (**a**) brushed sample (SZ), (**b**) non brushed sample (NSZ). The error bars show the standard deviation (series size n = 12). The tables below summarise the relative change in the flexural strength Δσ_f_, %, during exposure time (T_1_ − T_0_) (T_2_ − T_1_) and (T_2_ − T_0_) calculated according to Formula (5). Statistically insignificant differences Δσ_f_ are highlighted in grey.

The analysis of F-exposure’s influence on the flexural strength revealed a decrease for SZ series samples. No decrease was reported for NSZ samples. Still, it should be emphasised that a statistically significant change in the strength occurs already after the first exposure period T_1_, which lasts 336 h for F-exposure and 300 h for X-exposure. Further exposure up to 2016 h does not cause a significant change in the strength (Figure 14).

Different behaviour of SZ and NSZ profiles during F-exposure, where each weekly cycle starts with a 24 hours’ phase of wetting through condensation (Table 2), can be explained by the difference in the surface’s condition. As demonstrated in a previous study, mechanically treated profiles can be more susceptible because of lignocellulosic fibres’ exposure in the process [7,28,31]. It is assumed that the exposed hydrophilic fibres swell due to their wetting, which weakens the interaction forces between the matrix and the filler and deteriorates the strength [20,53]. A similar effect was observed for planned profiles made of HDPE composite with a wood flour filling [30].

X-exposure did not deteriorate the flexural strength of either SZ or NSZ series samples (Figure 14). It can be concluded that short-term spraying used in the exposure, followed by long-term light exposure combined with an elevated temperature (Table 2), does not exert such a significant influence on the NFPC’s strength as the exposure including long-term wetting. The results after X-exposure can even suggest that exposure to elevated temperature (BST 60 °C—see Table 2) could result in plastification of the polymer matrix and its better surrounding by the filler, and hence improvement in the interphase bonds [48]. A decrease in the flexural strength after X-exposure was observed in most of the previous papers, reaching 20–25% [27,30,31]. Still, it has to be pointed out that most of the papers concerned composites with HDPE matrix, which is less resistant to UV than PVC [13,32]. A lack of significant changes in the flexural strength corresponds to the results of brittle fracture analysis, which did not reveal any changes in the composite’s microstructure as a result of weathering (Figure 8).

**Figure 15:**
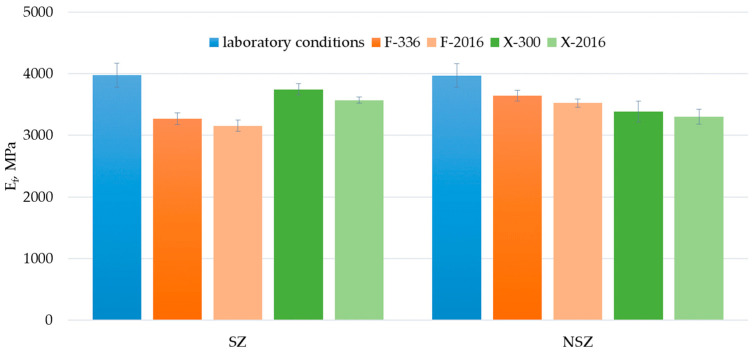
Results of flexural modulus tests of brushed (SZ) and non-brushed (NSZ) samples in basic state (laboratory conditions), after F-exposure for 336 h (F-336) and 2016 h (F-2016) and after X-exposure for 300 h (X-300) and 2016 h (X-2016). The error bars represent the standard deviation (series size n = 12).

Flexural modulus is another mechanical property analysed in the study. It depicts the material’s stiffness, which is a key feature for construction products installed with point support, e.g., on a grid, as happens with terrace and facade profiles [13]. The flexural modulus’ value level determines the profiles’ susceptibility to deformation under service loads [22]. The solutions examined in the study achieved the flexural modulus values of 3970 MPa in the original condition (Figure 15). As shown in a previous study, construction profiles made of composite with HDPE matrix and wood flour are characterised by the flexural modulus of 2530 ÷ 3600 MPa [36,38]; with PP matrix and wood flour—ca. 4500 MPa [54]; and with HDPE matrix and sisal and bamboo fibres—2500 MPa and 3700 MPa, respectively [52].

**Figure 16:**
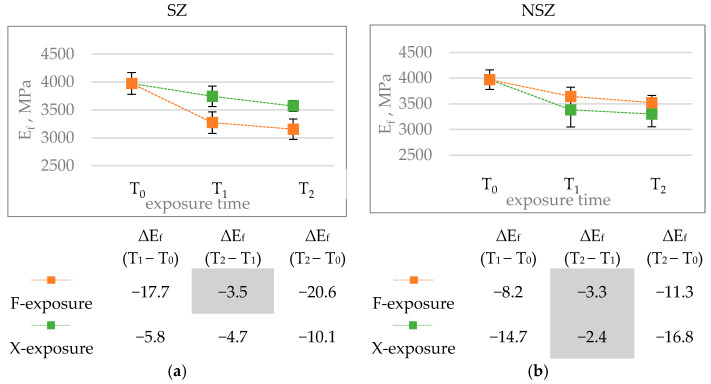
Diagrams showing differences in the flexural modulus E_f_, MPa, after F-exposure and X-exposure for time T_1_ and T_2_: (**a**) brushed sample (SZ), (**b**) non brushed sample (SZ). The error bars show standard deviation (series size n = 12). The tables under the diagrams summarise the relative change in the modulus of elasticity ΔE_f_, %, for exposure time (T_1_ − T_0_) (T_2_ − T_1_) and (T_2_ − T_0_), calculated according to Formula (5). Statistically insignificant differences ΔE_f_ are highlighted in grey.

The performed ageing procedures exerted a significant influence on the flexural modulus values (Figure 16). A decrease was observed in all tested series after the exposure. Similarly to flexural strength, increasing the time from 336 h for F-exposure and 300 h for X-exposure to 2016 h does not cause a statistically significant difference in the flexural modulus. The difference in the modulus of elasticity between SZ-X-300 and SZ-X-2016 is the exception for which the changes are noticeably lower than after 300 h of X-exposure.

The influence of F-exposure on brushed profile samples was most significant. The modulus of elasticity amounted to 3270 MPa (SZ-F-336) and 3160 MPa (SZ-F-2016) after F-exposure. For unbrushed samples, the values reached 3640 MPa (NSZ-F-300) and 3520 MPa (NSZ-F-2016). Similarly to flexural strength, exposing the lignocellulosic fibres during brushing could play a decisive role [7,31]. The reduction in the interphase interaction on the composite’ surface can determine the value of the modulus of elasticity much more than the flexural strength. Weakening of the top layer significantly increases susceptibility to strain [9,53]. Moreover, X-exposure reduced the modulus of elasticity’s value, whereby the non-brushed sample series revealed more significant differences than the brushed ones. The results correspond to the results of experiments performed for profiles with HDPE matrix and wood flour filling [28], although the drops discovered in this study are much smaller.

**Figure 17:**
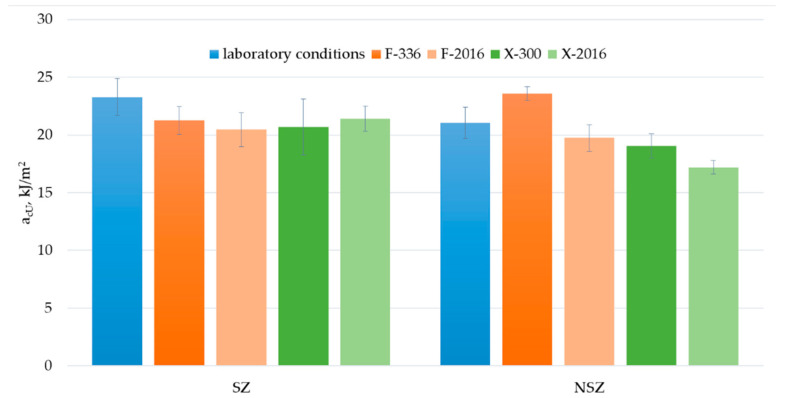
Results of Charpy impact strength of brushed (SZ) and non-brushed (NSZ) samples in basic state (laboratory conditions), after F-exposure for 336 h (F-336) and 2016 h (F-2016) and after X-exposure for 300 h (X-300) and 2016 h (X-2016). The error samples represent the standard deviation (series size n = 8).

Charpy impact strength is another mechanical property taken into account in the study (Figure 17). Because of the high risk of construction profiles’ exposure to dynamic loads throughout their entire life, stable impact strength value expressing the material’s susceptibility to fracture can be considered one of the key functional parameters.

The difference between the samples’ impact strength before and after F-exposure and X-exposure seems significant, but because of the dispersion of the results in each test series, the statistical significance for some of these changes cannot be confirmed. It applies, especially to SZ samples. An anomaly is observed for NSZ samples, involving a significant increase in the impact strength after F-336 exposure. After F-2016 exposure, the impact strength decreases significantly compared to F-336 exposure. The final impact strength change between the initial value and the value after F-2016 exposure is not statistically significant, although it amounts to over 6%.

**Figure 18:**
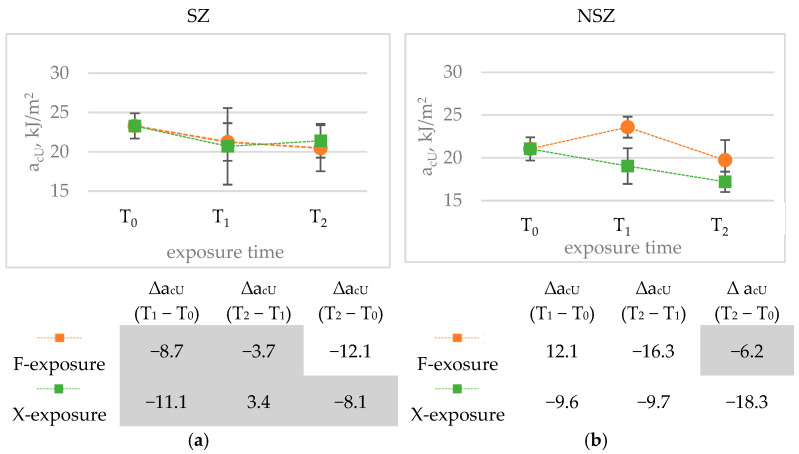
Diagrams presenting differences in Charpy impact strength, a_uC_, kJ/m^2^, after F-exposure and X-exposure for time T_1_ and T_2_: (**a**) brushed samples (SZ), (**b**) non brushed samples (NSZ). The error bars represent the standard deviation (series size n = 8). The tables under the diagrams present the relative change in the impact strength Δa_cU,_ %, for exposure time (T_1_ − T_0_) (T_2_ − T_1_) and (T_2_ − T_0_), calculated according to Formula (5). Statistically insignificant differences Δa_cU_ are highlighted in grey.

The general trend observed for the change in the mechanical properties after weathering (Figure 14, Figure 16 and Figure 18) is a statistically significant change after weathering time T_1_. However, in most cases, the difference between T_1_ and T_2_ is minor. The course of the Charpy impact strength changes for NSZ under F-exposure is the only exception.

In order to comprehensively evaluate the exposure type influence on the change in the mechanical properties Δ*Y*, the following equation was used:

(7) $$\Delta Y=100\%\cdot \frac{Y\left(F,T_{j}\right)-Y\left(X,T_{i}\right)}{Y\left(T_{0}\right)}$$

where: *F*, *X*—exposure type (according to Table 2), *Y*(*F*,*T_i_*), *Y*(*X*,*T_i_*)—value of the mechanical property after F- and X-exposure in time *T_i_ T_j_*^.^ Exposure times: *T*_0_—zero hours (before exposure), *T*_1_—336 h for F-exposure and 300 h for X-exposure, *T*_2_—is 2016 h for both exposures.

The analysis results of the exposure type’s influence on the property changes are summarised in Table 5. All applied exposure types and their times are compared.

The analysis of the data summarised in Table 5 indicates that in five cases (except for the impact strength for SZ samples), the difference between the mechanical properties after short-term F-exposure and X-exposure (F-336 and X-300) and after long-term F-exposure and X-exposure (F-2016 and X-2016) is statistically significant. The mechanical properties of the SZ surface material revealed the highest drop after F-exposure (negative values in the Table), while for the NSZ surface, it was after X-exposure (positive values in the Table). Hence, it can be concluded that for the SZ surface, more unfavourable changes can be expected after F-exposure, while for NSZ surfaces, it occurs after X-exposure. Both the exposure type and duration do not significantly contribute to the change in the Charpy impact strength for SZ samples.

On the other hand, an absence of a significant difference between the mechanical properties after F-exposure for 2016 h and X-exposure for 300 h can be observed. The modulus of elasticity for SZ samples makes the only exception, where significant differences in the modulus’ value can be observed between all exposure types. Major changes in the modulus (lower modulus values after exposure) are caused by F-exposure, while ranking the mechanical property values after both exposures (from the highest to the lowest value of the modulus), we get X-300; X-2016; F-336 and F-2016.

## 4. Conclusions

An analysis of the experimental data collected under the study suggests that exposing construction profiles made of PVC composite with wood flour filling to light emitted by different laboratory sources of light alternately with wetting causes degradation of their usable surfaces. An SEM analysis of the surface microstructure revealed molten areas in the polymer matrix outermost layers and exposed surfaces of the filler fibres. The degree of the changes can be considered as significantly reducing the profiles’ aesthetic and decorative properties. No microstructure changes were observed in the brittle fracture tests. However, the weathering procedure was discovered to impact the surface morphology. Influences involving irradiation with a xenon lamp and short-term wetting (X-exposure) caused much more significant surface degradation than exposure to fluorescent lamp’s light and long-term wetting (F-exposure) for the same exposure duration. The observations applied only to the mechanically non-treated usable surfaces. The observations were not carried out for brushed surfaces, because of the surface roughness.

The applied exposures affected the mechanical properties. The influence of surface treatment on changes in mechanical properties during weathering was not statistically analyzed due to the different mechanical properties of SZ and NSZ profiles before weathering. In the drawings, however, differences can be observed were observed in the susceptibility to the exposure for profiles with mechanically treated usable surfaces (brushed—SZ) compared to non-brushed (NSZ) ones. Artificial weathering carried out with fluorescent lamps and long-term wetting, included by condensation (F-exposure), greatly influenced the brushed profiles’ properties. In turn, the influence of artificial weathering by exposure to a xenon lamp and short-term wetting was more significant for the non-brushed profiles. Significant changes in the flexural modulus were observed after the exposures, especially after F-exposure. No significant decrease in the flexural strength occurred, and the impact strength changes are hard to assess because of the dispersion of the results in each series.

The exposure duration (time) affected the properties of interest. Although extending the weathering exposure time from 300 h (X-exposure) or 336 h (F-exposure) to 2016 h significantly aggravated the surface morphology changes, especially after X-exposure, the changes in the mechanical properties observed at the initial stage of ageing progressed only slightly.

The constancy of mechanical properties matters for the building fitness assessment. Based on the collected data, it can be concluded that for profiles made of natural fibre-reinforced polymer composites, whose usable surface was developed in a standard way, by mechanical treatment (brushing), artificial ageing using fluorescent lamps and long-term wetting including condensation (F-exposure) seems to be the adequate procedure to assess the changes in the mechanical properties. The procedure causes more severe swelling of lignocellulosic fibres and weakens the interaction forces between the matrix and the filler, reducing the mechanical parameters. The influence was particularly evident for the flexural modulus, which should be considered as a suggestion to select this parameter as a diagnostic feature of resistance to accelerated weathering. It needs to be emphasised that the changes in the modulus of elasticity can be determined only after short-term F-exposure (336 h), which can be used for quick diagnostics of new solutions.

This study does not exhaust the topic of artificial weathering resistance test methods for construction profiles made of plant fibre-reinforced polymer composites. Considering the dynamic development of this product group and its growing significance in civil engineering, further studies are planned. Future studies will cover other NFPC compositions and extended weathering exposure time.

## Figures and Tables

**Table 5:** Differences between the mechanical properties after F- and X-exposure in time T_1_ and T_2_ according to Equation (7), %. Statistically insignificant differences are highlighted in grey.

Changes in the Flexural Strength, %					
Samples SZ			Samples NSZ		
	X-300	X-2016		X-300	X-2016
F-336	−6.9	−7.6	F-336	4.7	4.0
F-2016	−6.4	−7.0	F-2016	3.8	3.0
**Changes in the** **Modulus of Elasticity, %**					
SZ samples			NSZ samples		
	X-300	X-2016		X-300	X-2016
F-336	−11.9	−7.5	F-336	6.5	8.6
F-2016	−14.8	−10.4	F-2016	3.5	5.6
**Changes in the Charpy Impact Strength, %**					
SZ samples			NSZ samples		
	X-300	X-2016		X-300	X-2016
F-336	2.4	−0.6	F-336	21.6	30.3
F-2016	−1.0	−4.0	F-2016	3.3	12.1

## Data Availability

The data presented in this study are available on request from the corresponding author.
